# Reduced Computed Tomography Scan Speed Improves Alignment Errors for Patients Undergoing Thoracic Stereotactic Body Radiation Therapy

**DOI:** 10.3390/cancers17162646

**Published:** 2025-08-13

**Authors:** Ramaswamy Sadagopan, Rachael M. Martin-Paulpeter, Christopher R. Peeler, Xiaochun Wang, Paige Nitsch, Julianne M. Pollard-Larkin

**Affiliations:** Department of Radiation Physics, The University of Texas M. D. Anderson Cancer Center, Houston, TX 77030, USA; rmmartin@mdanderson.org (R.M.M.-P.); crpeeler@mdanderson.org (C.R.P.); xiaochunw@mdanderson.org (X.W.); plnitsch@mdanderson.org (P.N.); jmpollard@mdanderson.org (J.M.P.-L.)

**Keywords:** SBRT, CT-on-rail, image guidance, motion amplitude, breathing frequency

## Abstract

**Objectives**: We investigated the performance of a slow computed tomography (CT) protocol to reduce alignment errors arising from motion when using CT-on-rail (CTOR) for image guidance for patients receiving thoracic stereotactic body radiation therapy (SBRT). **Methods**: A Quasar lung phantom with a moving tumor was programmed with three breathing rates and three motion amplitudes. MIP and average 4DCT images were used for contouring and alignment, respectively. Ten CTOR images were obtained for each of the breathing rates and amplitudes, under both CT protocols. We used in-house CAT software for image guidance, centering the tumor in the lung window within the gross tumor volume contour. Longitudinal coordinate reproducibility was compared between the two protocols. We also retrospectively analyzed CBCT SBRT image guidance alignment data from 31 patients to evaluate the systematic error in the longitudinal direction between simulation and daily treatments. **Results**: The mean (standard deviation) alignments (mm) for the standard and slow CT protocol ranged from 0.7 (0.68) and 1.0 (0.0), respectively, for the 28 BPM breathing rate and 5 mm amplitude combination to 5.2 (2.0) and 1.6 (0.52) for the 8 BPM breathing rate and 15 mm amplitude combination. Our retrospective analysis of patient alignment data showed a notable systematic difference in the relative bone and gross tumor volume alignment between the simulation and daily cone beam CT datasets. The mean longitudinal difference was −0.19 cm (standard deviation, 0.17 cm; range, 0.28 cm to −1.14 cm). Therefore, the position of the vertebral body cannot be used as a surrogate for mean tumor position in the longitudinal direction. Longitudinal position must be accurately determined for each patient using multiple CT images. **Conclusions**: A slow CT protocol improved the alignment with slower breathing rates being more challenging. A 5 mm PTV is not sufficient for tumor motion greater than 9 mm. Averaging the coordinates from multiple CTOR images is recommended.

## Simple Summary

Four-dimensional computed tomography (4DCT) has been used to account for breathing motion during the simulation of both stereotactic body radiation therapy (SBRT) and non-SBRT treatments. However, 4DCT is often unavailable, and it is impractical for image guidance. Due to its inherent slow acquisition, cone beam CT (CBCT) captures average tumor volume during multiple breathing cycles. However, CBCT’s image quality for certain tumors, such as small ground glass lesions or liver lesions, is notably insufficient. While CT-on-rail is an excellent alternative to CBCT, it has motion artifacts during on-board alignment due to its fast acquisition speed. To our knowledge, our current work is the first to addresses the motion artifact problem by evaluating the use of a slow CT protocol in CT-on-rail image guidance for patients undergoing thoracic SBRT. We also suggest steps to improve the alignment accuracy when using slow CT.

## 1. Introduction

Stereotactic body radiation therapy (SBRT) delivers a higher dose per fraction in fewer fractions than conventional radiation therapy, thus enhancing the biological effective dose (BED). Higher BED improves local control but also increases the potential normal tissue toxicity [1]. Therefore, accurate delineation of tumor and image guidance is critical. Thoracic and gastrointestinal lesions are affected by breathing motion [2,3] and require soft tissue-based image guidance, such as cone beam computed tomography (CBCT), in-room CT, or magnetic resonance imaging. CBCT acquisition is limited by gantry speed, which is typically about a minute for a full rotation [4]. Thus, CBCT image quality suffers from motion artifacts and image degradation due to the presence of scatter [5,6]. However, this long acquisition time also allows tumor motion to be averaged over many breathing cycles during CBCT imaging, allowing for a straightforward comparison between the averaged CBCT image set and the reference 4DCT image set. Despite the low-quality images, CBCT is useful for image guidance in thoracic SBRT due to its high contrast between lesions and lung tissue.

In-room CT, such as CT-on-rail (CTOR), has a fast gantry speed of about 0.5 s and provides diagnostic-quality images, which are preferred for low-contrast lesions, such as ground glass lesions and some liver tumors, especially when motion is present [7,8]. These high-quality images are also useful for visualizing some critical structures in the abdomen (e.g., gastrointestinal luminal structures). However, owing to their shared longitudinal direction, CT gantry motion and tumor motion cause an interplay effect. Thus, comparing daily images with an average reference image becomes difficult. This interplay effect leads to alignment error in the longitudinal direction, with the magnitude of the error depending on the breathing amplitude and frequency.

Thoracic and abdominal tumors are known to move when a patient breathes, posing additional challenges for imaging compared to immobile tumors. Before 4DCT became available, slow CT [9,10] was used to assess motion and delineate the internal gross tumor volume (iGTV) and normal structures. Previously, the limitations of slow CT for proper target coverage have been explored. Nakamura et al. [10] found that using only slow CT to acquire planning images was associated with a lack of target coverage, when tumor motion was <8 mm. Jang et al. [11] highlighted the advantage of using slow CT in combination with 4DCT. Currently, 4DCT-based simulation and CBCT-based image guidance are prevalent, which has made slow CT somewhat clinically irrelevant. However, 4DCT is not always available for in-room image guidance, and as noted above, CTOR is sometimes preferred over CBCT [12].

In our center, most patients who require thoracic SBRT undergo CBCT for image guidance. However, it is desirable to have diagnostic-quality images, such as those achieved with magnetic resonance imaging or CTOR, for image guidance purposes for difficult-to-view targets and critical structures. In addition, CT images are useful for adaptive planning, especially in proton and heavy ion treatments, where CT images can be used to verify the ranges of the beams [12,13]. Some centers, including ours, have elected to use CTOR-like platforms or mobile CT units for image guidance, as 4DCT is unavailable for image guidance, while others elected to implant fiducials in the target [14]. (We have a GE CTOR on two of the accelerators at our institution, and 4DCT is not available on these units.) Diagnostic CT protocols, such as those used for CTOR, use fast gantry speeds and high pitch to minimize motion artifacts. Hence, it is desirable to use motion management techniques, such as breath hold, to minimize the interplay between tumor motion and CT motion. However, in practice, using the breath hold technique is not always possible due to clinical and or practical reasons, such as duty cycle, patient condition, and highly variable tumor position even with the breath hold technique. In these impractical situations, a slow CT protocol could be used to reduce the interplay effect between the CT gantry motion and tumor motion.

In this work, we compared alignment inaccuracies of a standard CT protocol to those of a slow CT protocol and explored the usefulness and limitations of a slow CT protocol in image guidance using phantom studies. We also evaluated the impacts of the amplitude of tumor motion and breathing period on alignment accuracy, reporting the systematic error from the mean tumor position caused by the interplay effect between tumor motion and CT motion as a function of breathing amplitude and breathing frequency and providing some guidelines for patient selection. To illustrate the impact of ignoring the systematic errors during image guidance, we also quantified the observed systematic difference in the tumor alignment between simulated and daily CBCT imaging in 31 patients who underwent thoracic SBRT. Of note, we aimed to assess and minimize alignment errors, rather than study the dosimetric impact of alignment errors.

## 2. Methods

### 2.1. Phantom Measurements

**Figure 1:**
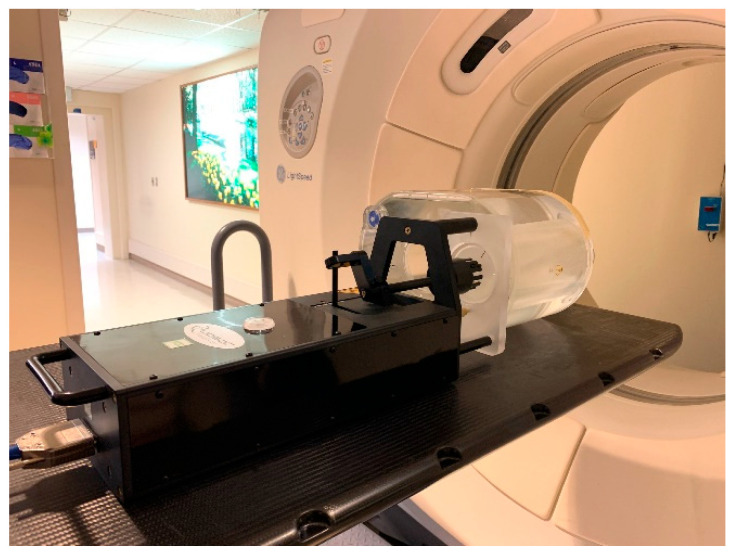
Phantom setup with motion accessories.

**Figure 2:**
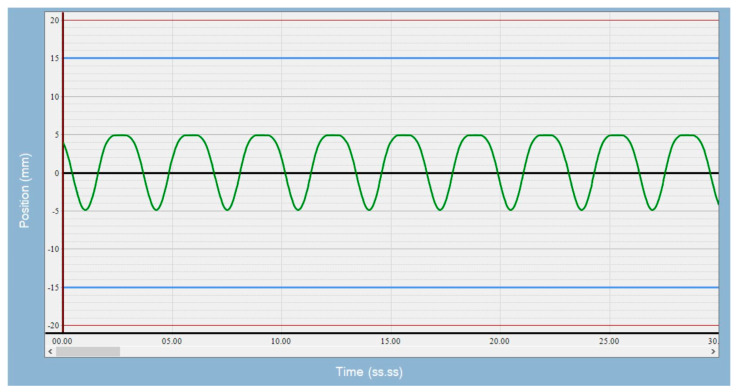
Sample of the motion waveform for 20 breaths per minute (BPM) used with the Quasar phantom.

We set up the Quasar Respiratory Motion Phantom (Modus Medical Device, London, ON, Canada) inside an air space, which was a substitution for a lung (Figure 1), and used a 3 cm-diameter sphere as the target. We were not concerned with the density difference between air and lung tissue, as we were interested in alignment errors, not the dosimetric impact of the alignment errors. We acquired a 4DCT image of the phantom on a Philips CT scanner (Brilliance Big Bore, Philips Healthcare, Andover, MS, USA) with a pressure sensing belt wrapped around the piston mechanism, so a breathing signal could be obtained to generate 4D images. In concordance with clinical practice, a series of 10 phase images was generated for each protocol; maximum intensity projection and average intensity projection images were derived from phase images and sent to the treatment planning system. Slice thickness was 2.5 mm, and pixel size was 0.96 mm for patient images and phantom images. The average intensity projection image was used as a reference image during image guidance, and the maximum intensity projection image was used to derive iGTV. Tumor motion amplitudes of 5, 10, and 15 mm and breathing rates of 8, 20, and 28 breaths per minute (BPM) were used for the simulation. Breathing motion is predominantly in the longitudinal direction. Since the CTOR gantry also moves in the longitudinal direction, there is an interplay between these two motions. The minor motions in the lateral and anterior posterior directions are captured in the axial images, and any motion artifacts if present can be visually averaged during alignment. Therefore, we focused this study on alignment errors due to longitudinal motion. A sample of the waveform used is shown in Figure 2. The top of the waveform represents the exhale phase, and the bottom of the waveform represents the inhale phase.

The phantom was set up on the CTOR unit table, and 10 images were obtained for each protocol (standard CT and slow CT protocols), as described below. Scan direction was alternated from head to feet and feet to head for successive scans. In total, 180 images were obtained across the three motion amplitudes, three breathing rates, and two CT protocols. The standard CT protocol had a rotation time of 0.5 s and pitch of 1.75:1, and the slow CTPITC had a rotation time of 1 s and pitch of 0.562:1. The standard protocol had a scan time of 9.9 s, and the slow protocol had a scan time of 59.5 s for the same scan length. The mA for the slow CT protocol was reduced, so that doses are comparable between the two protocols.

**Figure 3:**
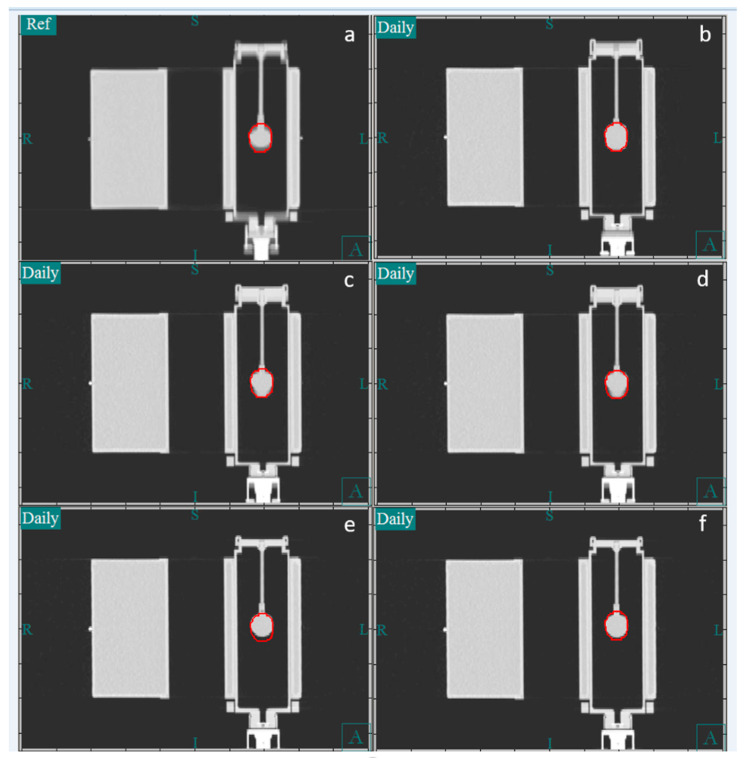
Images of the Quasar phantom with insert moving with an amplitude of 10 mm and breathing rate of 8 BPM. The red contour indicates GTV. (**a**) Reference average computed tomography (CT) image. (**b**) A slow CT image in which there was no difference between the bone and gross tumor volume (GTV) alignment. (**c**,**e**) Bone alignment for slow CT and standard CT, respectively. (**d**,**f**) GTV alignment for slow CT with a 2 mm shift and standard CT with a 4 mm shift, respectively. Notably, more of the respiration cycle was captured with slow CT compared to standard CT, leading to the blurred-out targets in the slow CT images. Standard CT was captured during peak expiration, leading to a relatively large error in alignment.

Image guidance was performed using our in-house software (CAT [15,16]). Figure 3 shows the CT image of the phantom in the coronal plane. Panel a shows the reference image, which is the average image of the 4DCT simulation. Panels b and c show a CT image obtained using the slow CT protocol, whereas panels e and f show one obtained using the fast CT protocol. In our clinical practice, during image guidance, the bone-based alignment to the vertebral body is compared to the soft tissue-based alignment to the iGTV to assess the reproducibility of the alignment. Because the motion phantom lacks vertebral bodies, the external surface of the phantom was used as a surrogate for the vertebral body. For soft tissue alignment, the GTV was manually centered within the center of the iGTV contour. The vertical, longitudinal, and lateral couch coordinates were recorded for the bone and iGTV alignments. Because the tumor in the phantom moved only in the longitudinal direction, the lateral and vertical directions were not reported.

### 2.2. Patient Measurements

During image guidance for SBRT, it is our practice to assess tumor drift over time. We compare bone and iGTV alignments using daily CBCT (or CTOR) and deem the tumor stable if the difference between two alignments is less than 5 mm in any cardinal direction. On the first day of treatment, if the difference between alignments using vertebral bone, and iGTV is 5 mm or more, then a second CT or CBCT image is obtained. To proceed with treatment, the difference between bone and iGTV must be consistently less than 5 mm between the first and second CBCT images. If these conditions are not met, a third CT or CBCT is obtained. If the difference is now less than 5 mm, the treatment is allowed to proceed. In the case of CBCT, the alignment value from the most recent image is used for treatment, but in the case of CTOR, an average alignment value from all images is typically used.

If the mean tumor position with respect to bone on the average CT image remains stable between the simulation and daily treatment, the vertebral longitudinal alignment can be used to reduce the interplay between tumor motion and the CT gantry translation. We investigated the stability of the relationship between bone and mean tumor position by retrospectively analyzing treatment data for consecutive patients who underwent thoracic SBRT for lung tumors from 1 March 2022 to 31 May 2022. Overall, 31 patients were included in the analysis. If a patient had multiple CBCT images, all CBCT images were included in the analysis. For this analysis, we considered only patients who underwent daily CBCT imaging and excluded patients who underwent CTOR, because we wanted to evaluate the systematic error in the longitudinal direction (i.e., error in the absence of the interplay between gantry and tumor motion). Additionally, we excluded patients who had undergone treatment with breath hold. First, bone-based alignment was completed using the vertebral bodies at the same level as the GTV. The shifts in the three cardinal directions were recorded. Soft tissue-based alignment was then completed. The difference between bone-based vertebral body alignment and soft tissue-based GTV alignment is a systematic error and provides a means to evaluate the robustness of the reproducibility of the patient setup between the simulation and daily treatments. Because CBCT requires a full minute for image acquisition, the image represents the average position of the GTV over several breathing cycles and, therefore, is a more accurate measure of the systematic error compared to the average position of the GTV determined by CTOR, which catches the GTV in snapshots during random breathing phases.

### 2.3. Statistical Analysis

Statistical testing was performed to assess the significance of the differences in mean absolute target displacements for the standard and slow CT protocols for the 3 levels of target displacement (5 mm, 10 mm, and 15 mm) for the 3 breathing rates (8 bpm, 20 bpm, and 28 bpm). R (version 4.5.1, R Core Team, Vienna, Austria) was used to perform two-sided Welch’s *t*-tests to calculate *p*-values for all comparisons. Significance was assessed at the 95% confidence level.

## 3. Results and Discussion

The longitudinal alignment of the Quasar motion phantom image data, including the mean and standard deviations in all 10 repeats of the alignments, using both protocols on the CTOR unit are presented in Table 1. For the 28 BPM breathing rate and the 5, 10, and 15 mm motion amplitudes, the systematic longitudinal alignment error ranged from 0.7 mm to 2.1 mm (standard deviation [SD] 0.68 mm to 1.60 mm) for the standard CT protocol and 0.9 mm to 1.6 mm (SD 0.0 mm to 0.85 mm) for the slow CT protocol. For the 20 BPM breathing rate and the 5, 10, and 15 mm motion amplitudes, the systematic longitudinal alignment error ranged from 1.6 mm to 3.2 mm (SD 1.6 mm to 2.1 mm) for the standard CT protocol and 1.3 mm to 1.6 mm (SD 0.52 mm to 1.27 mm) for the slow CT protocol. For the 8 BPM breathing rate and the 5, 10, and 15 mm motion amplitudes, the systematic longitudinal alignment error ranged from 1.7 mm to 5.2 mm (SD 1.25 mm to 2.0 mm) for the standard CT protocol and 1.3 mm to 2.0 mm (SD 0.95 mm to 1.7 mm) for the slow CT protocol. The SDs for the slow CT protocol were consistently lower than those for the standard CT protocol, and the systematic longitudinal alignment errors were generally higher for the standard CT protocol compared to the slow CT protocol. Both the systematic error and the SD increased with increased amplitude and with decreased breathing rate.

Planning target volume (PTV) margin is dominated by systematic uncertainty, and random errors tend to average out [17]. Using the margin calculation suggested by Van Herk [17], we estimated that a systematic uncertainty of 2.64 mm and a random uncertainty of 2 mm would lead to a PTV margin of 5 mm. The systematic and random uncertainties measured in the alignments are listed in Table 1. Since the systematic and random uncertainties do not include patient-related uncertainties, we estimated the overall systematic uncertainty to be the sum of squares of both alignment-related and patient-related uncertainties. The required PTV margin was then estimated using the following equation, using an estimate of 2 mm for patient-related systematic uncertainty:

PTV (mm) = 2.5 × sqrt (2^2^ + ∑^2^) + 0.7 × σ − 3 (1)

where ∑ and σ values from Table 1 were used to calculate the PTV margins that are listed in Table 2 and correspond to systematic and random uncertainties due to phantom setup. The value 2 represents systematic uncertainty in the system and includes table and image resolution.

A systematic difference of 2 mm was achievable when using the slow CT protocol across all combinations of motion amplitudes and breathing frequencies, whereas this difference was not achievable using the standard protocol (Table 1). The slow CT protocol yielded a PTV margin of a maximum of 5.3 mm in all cases, but the standard protocol required a PTV margin of 10.3 mm at the 10 mm motion amplitude and 12.3 mm at the 15 mm motion amplitude when the breathing rate was 8 BPM.

**Figure 4:**
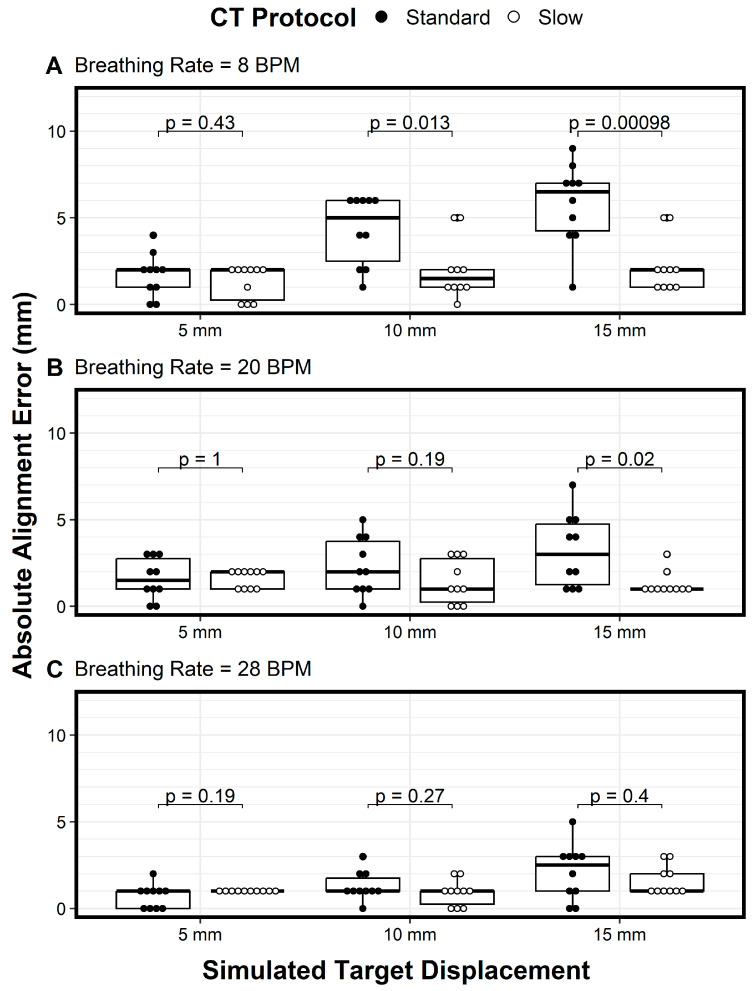
The absolute alignment error of the target is displayed with respect to simulated target displacements (amplitude of motion of the target) of 5 mm, 10 mm, and 15 mm for both standard CT and slow CT protocols using breathing rates of (**A**) 8 BPM, (**B**) 20 BPM, and (**C**) 28 BPM. Box-and-whisker plots indicate the median (solid center line), interquartile range (box), largest values within 1.5 times the interquartile range (whiskers), and outliers. Results of two-sided Welch’s *t*-tests comparing mean absolute alignment error for the two CT protocols are displayed as *p*-values for all levels of simulated target displacement for all breathing rates. *p*-values are displayed with two significant digits unless there was only one significant digit.

We plotted the variations of the longitudinal alignment for both protocols as a function of motion amplitude (displacement) when using breathing rates of 8 BPM (Figure 4A), 20 BPM (Figure 4B), and 28 BPM (Figure 4C). The Y axis denotes the alignment error, and the X axis denotes the motion amplitude. At a breathing rate of 20 BPM, the alignment error increased as the motion amplitude increased, especially for the standard CT protocol (Figure 4B, solid circles). In addition, the variation in the alignment error also increased as the motion amplitude increased for both protocols, indicating the need for more imaging samples with larger motion amplitudes (Figure 4B). These trends—the increase in alignment error and the increased variation in the error as the motion amplitude increased, for both the standard CT protocol and the slow CT protocol—were also observed for the 8 BPM and 28 BPM breathing rates (Figure 4A and Figure 4C, respectively). However, the magnitude of the alignment errors and the variation in the errors decreased as breathing frequencies increased (Figure 4A–C). Across all comparisons of the two protocols, the only differences in mean absolute alignment error that were statistically significant (*p* < 0.05) were for a breathing rate of 8 bpm with target displacements of 10 mm and 15 mm and for a breathing rate of 20 bpm with a target displacement of 15 mm.

Across all motion amplitudes and breathing rates, the slow CT protocol (open circles) had lower variation in alignment errors compared to the standard protocol (solid circles), with a general trend of variation increasing as breathing frequency decreased (Figure 4). These results show that the slow CT protocol reduced not only alignment errors but also variation in the alignment errors, thus requiring fewer imaging samples. For amplitudes of 10 mm and above, the alignment error variation was large enough to question the use of the standard PTV margin of 5 mm. For these cases, 4DCT during image guidance would be beneficial, and if 4DCT is not available, then slow CT with the slowest gantry or table speed should be used. However, using 4DCT for daily alignment would lengthen the procedure time and increase the radiation dose to the patient.

One way to address the effects of interplay effects on longitudinal alignment accuracy between the gantry and tumor motion is to use the mean tumor position of the planning 4DCT. This can be carried out by aligning the vertebral body between the planning 4DCT and CTOR. However, this assumes that the mean tumor position does not change from simulation to treatment or from one day to the next. This assumption was tested using retrospective study of alignment data from the 31 patients that were sequentially treated using CBCT. Our analysis showed a notable systematic difference in the relative bone and GTV alignment between the simulation and daily CBCT datasets. The mean ± SD difference in the longitudinal direction was −0.19 cm ± 0.17 cm (range 0.28 cm to −1.14 cm). Therefore, effort must be made to determine longitudinal position accurately for each patient with multiple CT images. Molitrois et al. [18] also came to the similar conclusion stating 5–7 mm variation between vertebral body and GTV.

Because the radiation dose is similar between the slow CT and standard CT protocols, the high contrast resolution and soft tissue contrast-to-noise ratio is similar between the two protocols. Image quality from CT images obtained with the slow CT protocol was not much different from the quality of CT images obtained with the regular CT protocol in our study. In addition, the tube current during the slow CT protocol can be increased as needed. Both slow CT and regular CT protocol have motion artifacts, but the image obtained with the slow CT protocol will have more blurring compared to the image obtained with the standard CT protocol. However, this blurring is desirable, because the target extent will be closer to that present in the average planning CT.

**Figure 5:**
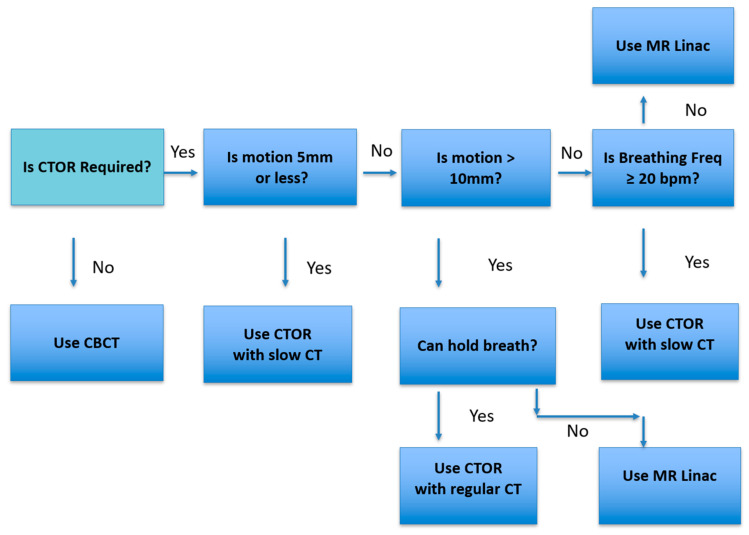
Flow chart illustrating image guidance technology selection in practice.

Figure 5 represents the workflow we use in our practice. When the lesion or critical structure visibility is poor, CTOR is considered for image guidance. Then lesion motion amplitude and breathing frequency are assessed. If the motion is 5 mm or less, or if it is under 10 mm and the breathing frequency is 20 bpm or greater, then CTOR is chosen; otherwise, MR Linac is chosen.

It is important to realize that this study only considers longitudinal motion. Though longitudinal motion is dominant, motion in another direction may be present and should be accounted for visually. Compared to patient anatomy, the phantom used does present some limitations in representing what would be encountered for actual treatment. The phantom materials do not perfectly mimic human tissue and anatomy, especially for tumors outside the lung, so the visibility of some targets and particularly target edges would be less sharp. Further study with a lower contrast phantom would be needed to confirm the results of this work apply in an abdominal setting. However, our clinical experience has shown that slow CT protocol is useful in the abdomen region, and images are similar to average CTs in the abdomen. Depending on the location of the target, it is possible that the motion could occur in multiple directions, as opposed to only the superior–inferior motion that was assessed in the study. We anticipate that motion in other directions could still be captured by the slow CT protocol. We elected to focus on superior–inferior direction motion for this study based on the rationale presented earlier in the Section 2 and our clinical experience.

## 4. Conclusions

Our study indicates that the slow CT protocol was more effective at predicting actual mean tumor location than the standard protocol. This finding has major implications on proper alignment with targets during image guidance. The alignment accuracy depends on not only the motion amplitude but also the breathing rate. Notably, a slower breathing rate was associated with higher alignment error. In the absence of 4DCT or CBCT, we recommend using a slow CT protocol with the lowest pitch possible for all patients requiring thoracic and gastrointestinal CT without breath holding. We recommend taking an even number of multiple CT scans, in alternating directions (cranial to caudal and vice versa), and averaging the longitudinal coordinates to determine mean target position more accurately.

A PTV margin of 5 mm is sufficient for the tumor motion measured in the current study if using the slow CT protocol. However, a PTV margin of 5 mm is not sufficient if using a standard CT protocol for tumor motion of 10 mm or more, especially for slower breathing rates. If acquiring a new CT scanner for image guidance, a 4DCT option is desired, along with image guidance software that can handle 4DCT images readily.

## Figures and Tables

**Table 1:** The mean (∑) and standard deviation of the 10 CT longitudinal alignments for various amplitude and BPM conditions for standard and slow CT protocols. Abbreviation: AVG, mean; stdev, standard deviation; STD, standard; SLOWCT, slow CT.

Motion Amplitude	CT Protocol	AVG (1 STDEV) (mm)		
		28 BPM	20 BPM	8 BPM
				
**5 mm**	**STD CT**	0.7 (0.68)	1.6 (1.60)	1.7 (1.25)
	**SLOWCT**	1.0 (0.00)	1.6 (0.52)	1.3 (0.95)
				
**10 mm**	**STD CT**	1.3 (0.83)	2.3 (1.64)	4.3 (2.00)
	**SLOWCT**	0.9 (0.74)	1.4 (1.27)	2.0 (1.70)
				
**15 mm**	**STD CT**	2.1 (1.60)	3.2 (2.10)	5.2 (2.00)
	**SLOWCT**	1.6 (0.85)	1.3 (0.68)	1.6 (1.69)

**Table 2:** Estimated PTV margins for various breathing rates and motion amplitudes. PTV, planning target volume.

Motion Amplitude	CT Protocol	PTV mm		
		28 BPM	20 BPM	8 BPM
				
**5 mm**	**STD CT**	2.8	4.5	4.4
	**SLOWCT**	2.6	3.8	3.6
				
**10 mm**	**STD CT**	3.5	5.8	10.3
	**SLOWCT**	3.0	4.0	5.3
				
**15 mm**	**STD CT**	5.4	7.9	12.3
	**SLOWCT**	4.0	3.4	4.6

## Data Availability

The phantom data was uploaded to the website zenodo.org. (https://doi.org/10.5281/zenodo.17162027).

## Abbreviations

The following abbreviations are used in this manuscript:

SBRT	stereotactic body radiation therapy
CBCT	cone beam computed tomography
4DCT	four-dimensional computed tomography
CTOR	CT-on-rail
IGTV	internal gross tumor volume
BPM	breaths per minute
PTV	planning target volume
MRL	magnetic resonance image guided Linac
