# Correction: Sadagopan et al. Reduced Computed Tomography Scan Speed Improves Alignment Errors for Patients Undergoing Thoracic Stereotactic Body Radiation Therapy. *Cancers* 2025, *17*, 2646

**DOI:** 10.3390/cancers18020184

**Published:** 2026-01-06

**Authors:** Ramaswamy Sadagopan, Rachael M. Martin-Paulpeter, Christopher R. Peeler, Xiaochun Wang, Paige Nitsch, Julianne M. Pollard-Larkin

**Affiliations:** Department of Radiation Physics, The University of Texas M. D. Anderson Cancer Center, Houston, TX 77030, USA; rmmartin@mdanderson.org (R.M.M.-P.); crpeeler@mdanderson.org (C.R.P.); plnitsch@mdanderson.org (P.N.);

## Missing Backmatter Statements

In the original publication [1], the Informed Consent Statement and the Data Availability Statement were not included. These statements are as follows:

**Informed Consent Statement:** Informed patient consent was not needed because the cases presented here are completely anonymized. Also, individual patient anatomical or tumor data is not reported—only that the vertebral body cannot be reliably used to assess the longitudinal coordinate of the mean tumor position.
**Data Availability Statement:** The phantom data was uploaded to the website zenodo.org. (https://doi.org/10.5281/zenodo.17162027).

The authors state that the scientific conclusions are unaffected. This correction was approved by the Academic Editor. The original publication has also been updated.
